# Notices

**Published:** 1873-01

**Authors:** 


### NOTICES.

HORACE GREELEY, the friend of man, is dead. His great heart overflowed with sympathy for all of human kind who needed help. There is perhaps not a man living on earth whose death would awaken such sincere regret and heartfelt sorrow, and there is not a Christian heart that beats which is not filled with thankfulness at the announcement that, just before he closed his eyes in death, he gave a look of recognition to those around him, and in cheerfulness repeated the beautiful verse of scripture—" I know that my Redeemer liveth ;" a knowledge worth more than all worldly gold and glory.

" HEALTH AT HOME, or Hall's Family Doctor," by the Editor of Hall's Journal of Health, 790 pages, 8vo., published by James M. Betts & Co., of Hartford, Conn. This is a subscription book, and cannot be had at any book store, only of agents who are making from five to thirty dollars a day. Active intelligent persons who desire agencies can address as above. Persons who wish single copies can send their names to R. Stephen Hall, 40 Broadway, New York, Room 44.

A PROPOSITION.—" The Christian Weekly " is $2 a year, published by the American Tract Society, 150 Nassau street, New York. It is most handsomely illustrated with beautiful and chaste engravings, and contains a large amount of religious, political and general reading ; all its selections are judicious and useful, and its editorials on all subjects are ably written.

The " New York Weekly Witness " is one dollar a year, and contains a large amount of valuable reading, general, moral and religious, with the latest mail and telegraphic news, with a judicious and valuable synopsis of the views of the press of the different political parties as does the " Daily Witness," which has been established by private enterprise as a religious daily two cent newspaper, which is receiving now, and will secure more largely, the advertising patronage of business men, good and true, who will thus indirectly aid in disseminating profitable reading at a low price to all classes ; reading which befits the parlors and the firesides of the cultivated and refined and good every where.

These two papers and the " Journal of Health," cost four dollars a year. The writer, on his own responsibility, offers them at three dollars for 1873. No such amount of unexceptionable reading can be had in any other direction on the subjects named for many times the price, and we should like to see one hundred thousand families accept the offer.

" The Servant-Girl of the Period ; the Greatest Plague of Life. What Mr. and Mrs. Honeydew learned of Housekeeping." By CHARLES CHAMBERLAIN, Jun. New York, J. S. Redfield, 140 Fulton street.

If there be any one subject which interests every family in the country, it is the subject-matter of this book. The annoyances we are all subject to in regard to our " help," at some period or other of our housekeeping experience, form a staple topic of conversation ; and in the volume before us, every housekeeper will be interested in the experiences of poor Mrs. Honeydew. The author has made a very clever and entertaining story, into which he must have woven a great many incidents from somebody's real life. The volume is crown 8vo., 216 pages. Price, paper, 75 cents, bound, $1.25.

PLEASANT REMINISCENCES of old 1872 must come trooping in on Baldwin, the Clothier, when he remembers that at his mammoth establishment on the corner of Broadway and Canal Street, New York, he has sold during the year past at retail $348,000 worth of Boys' Clothing alone, and altogether fourteen hundred and one thousand dollars—sold, paid over and received by him for clothing ; and what is astounding, without the loss of one dollar in bad debts, because he sells for cash at one price to friend and stranger alike. With him " business is business,"—one sale, one price, and that price cash down.

